# MicroRNAs as regulators and mediators of forkhead box transcription factors function in human cancers

**DOI:** 10.18632/oncotarget.14015

**Published:** 2016-12-16

**Authors:** Chen Li, Kai Zhang, Jing Chen, Longbang Chen, Rui Wang, Xiaoyuan Chu

**Affiliations:** ^1^ Department of Medical Oncology, Jinling Hospital, School of Medicine, Nanjing University, Nanjing, Jiangsu, PR China

**Keywords:** microRNA, forkhead box transcription factor, cancer, post-transcriptional regulation

## Abstract

Evidence has shown that microRNAs are widely implicated as indispensable components of tumor suppressive and oncogenic pathways in human cancers. Thus, identification of microRNA targets and their relevant pathways will contribute to the development of microRNA-based therapeutics. The forkhead box transcription factors regulate numerous processes including cell cycle progression, metabolism, metastasis and angiogenesis, thereby facilitating tumor initiation and progression. A complex network of protein and non-coding RNAs mediates the expression and activity of forkhead box transcription factors. In this review, we summarize the current knowledge and concepts concerning the involvement of microRNAs and forkhead box transcription factors and describe the roles of microRNAs-forkhead box axis in various disease states including tumor initiation and progression. Additionally, we describe some of the technical challenges in the use of the microRNA-forkhead box signaling pathway in cancer treatment.

## INTRODUCTION

The protein products of forkhead box (FOX) constitute an extended family of transcription factors characterized by the presence of an evolutionary conserved “forkhead” or “winged-helix” DNA-binding domain (DBD), a trans-activation or trans-repression effector region.[1, 2]. More than fifty five or fifty forkhead proteins have been identified in mammals or humans genome, respectively, and they are further classified into 19 subgroups (FOXA to FOXS) basing on sequence homology inside and outside the forkhead domain [3, 4]. Subclasses are designated by a letter, and genes within each subfamily are identified by an Arabic numeral. The typography follows the conventions: all uppercase letters for human (e.g., FOXB1); only the first letter capitalized for mouse (e.g., Foxb1); the first and subclass letters capitalized for all other chordates (e.g., FOXB1) [3–5].

**Figure 1:**
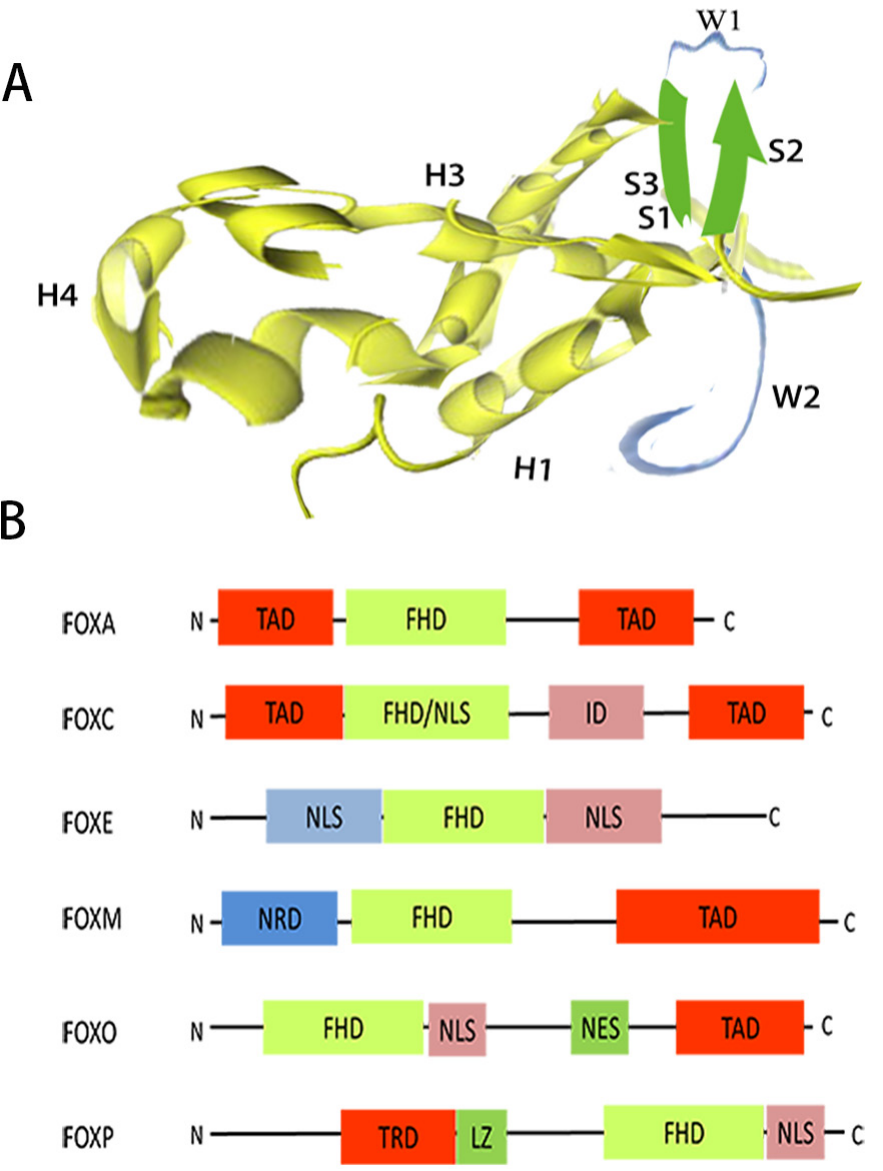
Structural organization of the FOX family **A**. Three-dimensional structure of the DNA-binding domain of FOXO4, showing helical (H) sections, β-strand(S) sections and winged (W) sections [8]. **B**. Schematic diagram of primary structures of different FOX proteins. ID, inhibitory domain; LZ, leucine zipper; NES, nuclearexport sequence; NLS, nuclear localization sequence; NRD, N-terminal repressor domain; TAD, transactivation domain; TRD, transcriptional repressor domain.

Burley et al worked out the first structure of a forkhead domain (FOXA3) by X-ray diffraction crystallography [6]. By comparing the fold with the shape of butterflies, they coined the term “winged helix” as nickname to describe the structure. All FOX proteins contain the characteristic 100-aminoacid winged helix/forkhead box domain (FBD/FHD), which defines this class of transcription factors [7]. As a compact structure, the FBD contains a helix-turn-helix core of three N-terminal a-helices (H1-3), three β-strands (S1-3), flanked by two loops (W1-2) towards its C-terminal region (Figure 1A) [7, 8]. FOX proteins are involved in chromatin remodeling and nuclear localization. DNA-binding affinity and specificity of the FOX transcription factors essentially involves the variable region at the junction of helices H2 and H3 and wings. Romanelli et al provided the first documentation about nuclear targeting of a forkhead protein (FOXE1) containing two nuclear localization sequences (NLS) signal flanking the DNA-binding domain [9]. The two identical bona fide NLSs are located at both ends of the FBD, one of which is located in H1 and the other of which is located in W2 [10]. Besides a highly conserved FHD and NLS located just downstream of FHD, molecules of different FOX proteins also have a nuclear export sequence, a transactivation domain, a transcriptional repressor domain, a leucine zipper or a inhibitory domain (Figure 1B) [4].

FOX proteins play pleiotropic roles in embryonic development and homeostasis of adult tissues due to the ability to coordinate temporal and spatial gene expression. They control cell fate decisions by regulating a wide spectrum of cellular processes including proliferation, cell cycle progression, differentiation, metabolism, migration, as well as apoptosis, survival, DNA damage response and drug resistance. Otherwise, the dysregulation and mutation of the super family FOX genes often induces human genetic diseases, including tumorigenesis [11]. Acting as transcriptional activators and repressors as well as pioneer factors, FOX proteins are found to be activated constitutively in several signaling pathways, such as Akt/ PKB pathway, TGF/β-Smad cascade, the Sonic-Hedgehog pathway and the Wnt/β-catenin pathway. A complex network of protein and non-coding RNAs including numerous miRNAs mediates the expression and activity of FOX transcription factors. More recently, a number of miRNAs have identified as regulators and mediators of FOX expression.

microRNAs (miRNAs), a conserved class of endogenous and noncoding small RNAs, regulate gene expression post-transcriptionally by binding to 3’-untranslated region (UTR) of target mRNAs, usually resulting in mRNA degradation or translational repression [12]. The first step in miRNA biogenesis is the formation of pri-miRNA transcribed by RNA Polymerase II in the nucleus [13]. Subsequently, this transcript is cropped into precursor miRNA by specific RNase III endonuclease DROSHA-double stranded RNA [14]. Then, the pre-miRNA is exported into the cytoplasm with the help of the nuclear export factor Exportin5 [15, 16]. Finally, the mature miRNA is generated by a second RNase III enzyme termed Dicer, followed by incorporation into the RNA induced silencing complex [17]. The guide strand of the duplex directly binds to an Argonaute protein, while the opposite strand (miRNA*) is often degraded and therefore not detectable [18]. Recently, miRNAs targeting transcription factors (TFs) have emerged as an important mechanism for gene expression regulation. The co-regulatory networks of TFs and miRNAs have been investigated to act either as buffers for gene expression or as quick repressive switches in a central hub [19, 20]. In this review, we summarize the current knowledge and concepts concerning the involvement of miRNAs and FOX transcription factors and describe the roles of the miRNA-FOX signaling pathway in various disease states including tumor initiation and progression. Additionally, we describe some of the technical challenges in the use of the miRNA-FOX signaling pathways in cancer treatment.

## FOX-RELATED MIRNAS

FOX transcription factors expression is often found to be activated constitutively in human cancers via several mechanisms, such as genetic mutation, chromosomal translocations, increased translation of FOX mRNA, loss of auto-regulation, or activating mutations in components of upstream regulatory pathways [21–23]. More recently, a number of miRNAs have been reported to regulate FOX transcription factors expression. Table 1 provides a list of miRNAs identified as regulators in the formation of FOX transcription factor isoforms, along with information on FOX-dependent biological processes. Meanwhile, several FOX transcription factors are also reported to regulate expression of miRNAs, and Table 2 provides a list of miRNAs regulated by FOX transcription factors. Given the importance of miRNAs and FOX transcription factors in tumorigenesis, targeting miRNA-FOX signaling pathways may a potential strategy for cancer therapy. Therefore, investigation of these signaling pathways is urgently needed.

**Table1:** Compilation of miRNAs targeting forkhead box family

FOX member		miRNAs		Diseases/Organ	Biological process	Reference
FOXA	FOXA1	miR-20a		Preeclampsia	proliferation, invasion	[27]
		miR-212		HCC	growth	[28]
	FOXA2	miR-124a		Pancreatic beta-cell	β-cell differentiation	[31] [22]
		miR-1291		Pancreatic cancer	proliferation,	[35]
FOXC	FOXC1	miR-204		Endometrial cancer	migration, invasion	[38]
				Trabecular meshwork	regulatory pathway	[40]
		miR-133		Pituitary tumor	migration, invasion	[39]
	FOXC2	miR-520h		Lung cancer	EMT	[48]
FOXF	FOXF2	miR-182		CRC	growth, invasion, metastasis	[157]
		miR-519		HCC	proliferation, apoptosis	[158]
FOXG	FOXG1	miR-9/33		Vertebrates	organ development	[161]
FOXJ	FOXJ2	miR-34a		EPCs	differentiation	[170]
	FOXJ3	miR-494		Skeletal muscle	mitochondrial biogenesis	[172]
		miR-27b		Myocytes	mitochondrial biogenesis	[173]
		miR-517		CRC	invasion, migration	[175]
				Lung cancer	proliferation, invasion	[174]
FOXM	FOXM1	miR-24-1		Bladder cancer	proliferation	[50]
		miR-149		CRC	migration, invasion	[53]
		miR-802		Breast cancer	proliferation	[51]
		miR-370		Gastric carcinogenesis	proliferation	[56]
				LSCC	proliferation	[55]
				CML	chemosensitivity	[54]
		miR-134		NSCLC	EMT	[58]
		miR-149		NSCLC	EMT	[59]
		miR-671		Breast cancer	proliferation, EMT	[52]
		miR-194		Gastric cancer	EMT	[57]
		miR-204		Esophageal cancer	invasion, EMT	[61]
		miR-29		AML	apoptosis	[62]
FOXN	FOXN1	miR-22		Keratinocyte Progenitor Cell	differentiation	[165]
		miR-205		thymus	T Cell Development	[166]
		miR-18b/518b		pluripotent cells	differentiation	[164]
FOXO	FOXO1 FOXO3	miR-96		CRC	proliferation	[71]
				HCC	proliferation	[75]
		miR-183/96/182		Granulosa cell	proliferation, cell cycle	[67]
				Breast cancer	proliferation, cell cycle	[68]
		miR-196a		HCC	proliferation	[70]
		miR-9		Leukemogenesis	myelopoiesis, differentiation	[76]
				Obese mice	gluconeogenesis	[69]
		miR-705		Osteoporosis	oxidative damage	[73]
		miR-137		HCC	polymorphisms	[72]
		miR-183		MREs	proliferation, migration	[74]
		miR-370		PC	proliferation	[90]
				Amputated fingers	proliferation	[91]
		miR-22		HEK293T	signaling kinetics	[103]
		miR-486		Chronic kidney disease	muscle wasting	[104]
		miR-145		HCC	growth	[105]
		miR-126		Mesothelioma	cancer progression	[107]
		miR-34a		EPCs	senescence, angiogenesis	[122]
		miR-146b		White adipose tissue	adipogenesis	[123]
		miR-132/212		FSH expression	GnRH activation	[124]
		miR-217		Metabolic disorders	EC senescence	[125]
		miR-339		Acupuncture	Acupuncture's effects	[127]
		miR-135b/194		HCC	proliferation, apoptosis	[77]
		miR-96		CRC	proliferation	[71]
				Breast cancer	proliferation	[80]
				Type I collagen matrix	IPF fibroblasts	[89]
				HCC	proliferation	[75]
		miR-9		Leukemogenesis	myelopoiesis, differentiation	[76]
		miR-223		Type I interferon production	antiviral innate immunity	[78]
		miR-592		CRC	tumorigenesis	[79]
		miR-153		CRC	invasion, drug resistance	[81]
		miR-182		Skeletal muscle	atrophy	[82]
		miR-1		Muscle	atrophy	[188]
	FOXO4	miR-421		NC	proliferation, apoptosis	[88]
		miR-23b		Vascular smooth muscle	phenotypic switching	[83]
FOXP	FOXP1	miR-34a		lymphoma	B cell progression	[130, 131]
		miR-150		CLL	growth, urvival	[132]
		miR-504		Glioma	proliferation, apoptosis	[136]
				OSCC	invasion	[135]
		miR-206		Cardiac hypertrophy	growth, survival	[133]
	FOXP2	miR-9/140		brain	speech and language	[140]
		miR-9/132		brain	radial migration of neurons	[139]
		let-7a/miR-9/129		brain	speech and language	[141]
		miR-199a		Breast cancer	metastasis	[144]
		miR-190		GC	growth and invasion	[145]
	FOXP4	miR-138		NSCLC	growth	[153]
		miR-338		HCC	growth	[154]
FOXQ	FOXQ1	miR-124		NPC	growth, metastasis	[177]
		miR-506		Cervical cancer	proliferation, EMT	[176]
				NPC	proliferation, invasion	[178]

Abbreviations: HCC, hepatocelluar cancer; CRC, colorectal cancer; EPCs, Endothelial progenitor cells; LSCC, laryngeal squamous cell carcinoma; CML, chronic myeloid leukemia; NSCLC, non-small cell lung cancer; AML, Acute myeloid leukaemia; MREs, miRNA recognition elements; GC, gastric cancer; PC, prostate cancer; NPC, nasopharyngeal carcinoma; CLL, chronic lymphocytic leukemia; OSCC, oral squamous cell carcinoma; UVECs, umbilical vein endothelial cells; EC, endothelial cell.

**Table 2:** Compilation of miRNAs regulated by forkhead box family members

FOX member	miRNA	Disease / Cell	Biological process	Reference
FOXM1	miR-135a	HCC	Tumor metastasis	[63]
FOXO1	miR-21	UVECs	EMT	[111]
FOXO3	miR-30d	RCC	apoptosis	[93]
	miR-21	CRC	growth	[115]
		Lung cancer	apoptosis	[113]
	miR-34b/34c	PC	EMT	[95]
FOXP3	miR-7/155	Breast cancer	Cancer progression	[147, 148]
	miR-155	T cell	Tumor proliferation	[149, 150, 189]
	miR-142-3p	T cell	cAMP	[152]

### FOXA subfamily and miRNAs

FOXA1 is a vital regulator of cell proliferation and migration, either during various normal organ development or cancer progression [24]. For example, the FOXA1-deficient mouse prostate shows a severely altered ductal pattern that resembles primitive epithelial cords rather than differentiated or mature luminal epithelial cells [25]. FOXA1 plays an oncogenic role and has been considered as a predictor of poor survival in hepatocelluar cancer (HCC), prostate cancer and breast cancer [26]. Investigators predicted miR-20a seed-matching sequences in the FOXA1 3’-UTR, and overexpression of miR-20a in human preeclampsia tissue was found to compromise the proliferative and invasive activities of trophoblast cells by repressing the expression of FOXA1 on mRNA and protein level [27]. Dou and his co-workers showed that miR-212 exerts its inhibitory effect on HCC by inhibiting FOXA1 expression via “seedless” 3’-UTR miRNA recognition elements [28].

**Figure 2:**
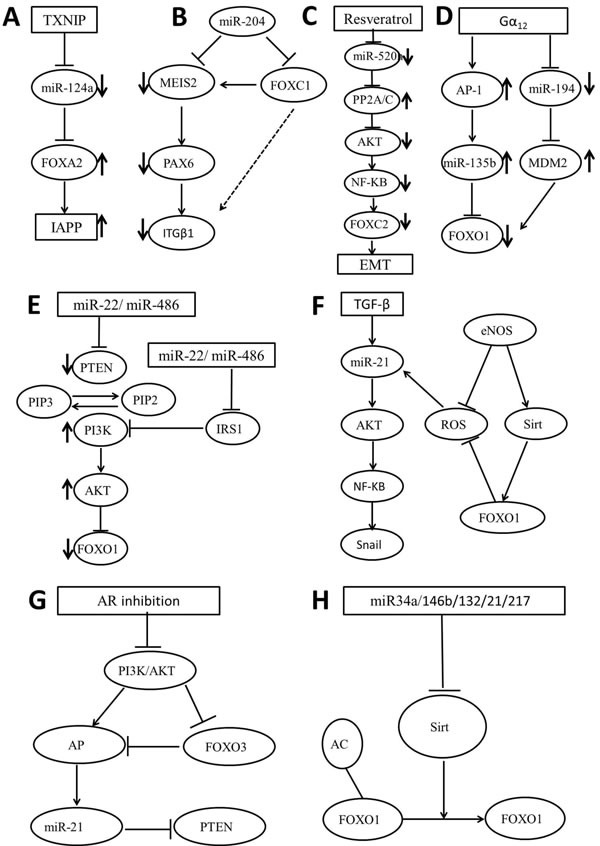
Schematic representation of the FOX regulating miRNAs signaling pathways **A**. TXNIP/miR-124a/FoxA2/IAPP; **B**. miR-204/MEIS2-FOXC1/PAX6/ITGβ1; **C**. resveratrol/miRNA-520h/PP2A/C/Akt/NF-κB/FOXC2; **D**. Gα12/AP-1/miR-135b/FOXO1-Gα12/miR-194/MDM2/FOXO1; **E**. miR-22-miR-486/PTEN/PI3K/AKT/FOXO1; **F**. TGF-β/FOXO1/miR-21/AKT/NF-KB/Snail; **G**. AR/PI3K/AKT/FOXO3/AP-1/miR-21/PTEN; **H**. miR-34a-146b-132-21-217/Sirt/FOXO1.

FOXA2 is an important regulator of pancreatic development. Also, FOXA2 is required for key β-cell-specific functions, stimulating the expression of many key genes involved in β-cell glucose sensing, such as pancreatic duodenum homeobox-1 [29, 30]. Moreover, FOXA2 contributes to insulin release via the regulation of genes coding Sur1 (sulfonylurea receptor 1) and Kir6.2 (inward rectifier potassium channel member 6.2), the two subunits of the ATP-sensitive K+ channel [30]. Recently, miR-124a was reported to directly or indirectly inhibit FOXA2 expression to function in β-cell differentiation [31]. Interestingly, thioredoxin-interacting protein (TXNIP) and islet amyloid polypeptide (IAPP) have also been shown to be up-regulated by glucose and diabetes and to be involved in β-cell apoptosis and inflammation [32]. Jing el al provided the first finding that TXNIP and IAPP are linked intricately by a transcriptional activation cascade [33]. Initially, TXNIP down-regulates miR-124a expression, whereas miR-124a overexpression leads to down-regulation of FOXA2 and IAPP promoter occupancy, then decreases IAPP expression, finally also effectively inhibits TXNIP-induced IAPP expression [33]. Taken together, a novel TXNIP/miR-124a/FOXA2/IAPP signaling cascade could provide a novel mechanistic insight into the transcriptional regulation in β-cell biology (Figure 2A). In addition to the previously described miR-124a, FOXA2 is also a direct target for miR-1291, a less studied suppressive miRNA which is generated from small nucleolar RNA H/ACA box 34 in pancreatic cancer cells [34, 35]. Meanwhile, miR-1291 modulates a lot of proteins to form a network of interactions in the control of cancer properties. Also, FOXA2 has been found to activate the transcription of AGR2 by binding the promoter region of AGR2 [36]. Based on these results, Tu et al connected miR-1291 to the FOXA2/AGR2 regulatory pathway in the suppression of pancreatic cancer cell proliferation.

### FOXC subfamily and miRNAs

Recently, FOXC1 has been reported to be a fundamental regulator in various aspects of human cancers including nasopharyngeal carcinoma [37]. It is reported that overexpression of miR-204 repressing migration, invasion and extracellular matrix-adhesion in endometrioid-type endometrial cancer cell line by targeting FOXC1 [38]. Moreover, miR-133 was found to inhibit pituitary adenoma cell invasion and migration and induce EMT by directly targeting FOXC1 [39]. Besides contributing to tumorigenesis, as a target of miR-204, FOXC1 was also confirmed in the trabecular meshwork related to the miR-204-MEIS2 (myeloid ecotropic viral integration site 1 homolog 2) regulatory pathway [40, 41]. MEIS2 is a homeobox transcription factor, which exerts multiple functions in vertebrate eye development and has been earlier identified as a miR-204 target [42]. Integrin β1 (ITGβ1) is expressed in various ocular tissues including the TM and regulated by PAX6, a master regulatory gene of eye development. In turn, PAX6 is also regulated by MEIS2 [43, 44]. As a result, FOXC1 exerts its indirect up-regulation of ITGβ1 via its effect on MEIS2 in the described regulatory pathway (Figure 2B).

Overexpression of FOXC2 correlates with tumor invasion and metastasis and is reported to promote EMT of tumor cells [45]. Activation of phosphatidylinositol 3-kinase (PI3K)/Akt has been reported to play an important role for FOXC2 expression in endothelial cells [46]. Moreover, PP2A/C, a catalytic subunit of serine/threonine phosphatase PP2A, has been reported to reduce Akt activity by dephosphorylating Akt [47]. Resveratrol is a polyphenol which inhibits several transcriptional factors associated with EMT including FOXC2 in lung cancer cells [48]. Based on the valid experimental data, Yu et al showed that resveratrol induces mesenchymal-epithelial transition and suppress tumor metastasis. It is firstly found that Resveratrol upregulates miR-520h-inhibited PP2A/C expression, consequently inactivates AKT/nuclear factor-κB (NF-κB) and finally inhibits the expression of FOXC2 (Figure 2C). These researchers provided a new insight into the role of resveratrol-induced molecular and epigenetic regulations in tumor suppression and provided new evidence supporting the therapeutic potential of resveratrol.

### FOXM subfamily and miRNAs

FOXM1 is a oncogenic transcription factor which is overexpressed in various types of cancer and implicated to have critical roles in cell migration, invasion, angiogenesis and metastasis [49]. Many studies have reported involvement of the miRNA-FOXM1 signaling pathway in different tumors. Recently, Inoguchi and his colleagues showed that downregulation of miR-24-1 in breast cancer cells leads to FOXM1 overexpression and promotes aggressive cancer behavior via regulation of p27^kip1^ degradation [50]. Also, overexpression of miR-802 suppresses breast cancer cell proliferation by downregulating FOXM1 [51]. Tan et al reported that miR-671-5p is a tumor-suppressor miRNA in breast tumorigenesis by directly targeting FOXM1, and also demonstrated that overexpression of miR-671-5p in breast cancer cells attenuates cell proliferation and invasion, induces S-phase arrest, inhibits EMT and sensitizes cancer cells to cisplatin, 5-fluorouracil and epirubicin exposure [52].

By gain and loss of function assays, miR-149 is found to be a negative regulator of migration and invasion of colorectal cancer cells via downregulation of MMPs, VEGF-A and uPAR, together by post-transcriptionally downregulating FOXM1 [53]. In another study, Zhou et al showd that downregulation of miR-370 expression is a frequent event in primary leukemic cells obtained from acute myeloid leukemia (AML) patients and upregulation of miR-370 inhibits proliferation and enhances chemosensitivity to Homoharringtonine of AML cells by by directly targeting the 3’-UTR of FOXM1 [54]. Likewise, FOXM1 is found to be directly downregulated by miR-370 in laryngeal squamous cell carcinoma [55]. It was reported that FOXM1 is overexpressed in Helicobacter pylori-induced gastric carcinogenesis and is negatively regulated by miR-370 [56]. Also, miR-194 inhibits the EMT phenotype in gastric cancer cells by targeting FOXM1 [57].

It has been shown that miR-134 and miR-149 are correlated with invasive potential and EMT phenotype in non-small cell lung cancer (NSCLC) cells by suppressing FOXM1 [58, 59]. In another study, miR-342-3p is reported to suppress proliferation, migration and invasion by targeting FOXM1 in human cervical cancer [60]. Another miRNA, miR-204 is also reported to inhibit invasion and reverse EMT in esophageal cancer by directly binding to the 3’-UTR of FOXM1 mRNA [61]. Also, miR-29 is observed to induce K562 cell apoptosis by downregulating FOXM1 [62]. Conversely, FOXM1 also regulates expression of miRNAs. For example, miR-135a is a FOXM1-induced miRNA which acts as an oncogenic miRNA in portal vein tumor thrombosis by repressing the expression of metastasis suppressor 1 [63, 64]. Another research showed that miR-135a, transcribed by FOXM1, contributes to the development of portal vein tumor thrombus by promoting metastasis in HCC by targeting metastasis suppressor 1 [63].

### FOXO subfamily and miRNAs

To date, four FOXO isoforms in humans (FOXO1, FOXO3, FOXO4 and FOXO6) have been identified, and all of them are expressed in most human tissues and usually regarded as tumor suppressors [65]. Posttranscriptional regulation of FOXO synthesis by various miRNAs has emerged as a new regulatory level of their functions. As demonstrated by numerous recent publications, a wide range of miRNAs regulate FOXO transcripts in longevity and age-dependent diseases, including cancer, neurodegenerative diseases and diabetes [66].

FOXO1 is also a target of many cancer-related miRNAs, including miR-96, miR-183-96-182 cluster, miR-196a, miR-9, miR-705, miR-137. These miRNAs targeting FOXO1 to exert their effects on cell apoptosis and proliferation in colorectal cancer, breast cancer and HCC [67–73]. Moreover, FOXO1 is regulated by miR-183 in human cells through the gain of a single nucleotide substitution, and this regulation is important for FOXO1-dependent functions [74]. A single miRNA might regulate several target mRNAs due to the relatively short seed region. For instance, FOXO1 and FOXO3a are both direct targets of miR-96 in colorectal cancer and HCC [71, 75]. Meanwhile, FOXO3 and FOXO1 are found to be critical targets of miR-9 in hematopoietic cells [76]. On the other hand, one FOX protein can be regulated by several miRNAs. Jung and his colleagues showed that miR-135b is markedly stimulated by Gα_12_ signaling in HCC cells through activator protein-1, which consistently leads to FOXO1 repression. In addition, Gα_12_QL represses miR-194 cluster gene products (194/192/215), which contributes to MDM2-mediated FOXO1 repression (Figure 2D) [77].

Different miRNAs have been found to be related with FOXO3 and FOXO4. For example, miR-223 promotes type I interferon production in antiviral innate immunity by targeting FOXO3 [78]. In another report, miR-592 promotes metastasis, in part, by targeting FOXO3A in human colorectal cancer [79]. Recently, miR-96 is found to directly bind to the 3’-untranslated region of FOXO3a mRNA, which subsequently inhibits its function [80]. Functional studies revealed that miR-153 upregulation increases resistance to Oxaliplatin and Cisplatin in colorectal cancer by inhibiting FOXO3a [81]. Researchers also demonstrated that miR-182 contributes to the regulation of FOXO3 by targeting FOXO3 in skeletal muscle during chronic diseases (e.g., diabetes, chronic kidney disease) which are associated with elevated glucocorticoid production [82]. By gain and loss of function, FOXO4 is testified as a direct target of overexpressed miR-23b in phenotypic switch of vascular smooth muscle cells and is involved in proliferation and migration of several non-vascular cell types [83].

.The PI3K/AKT pathway has emerged as an important fundamental pathway via phosphorylation of a variety of substrates [84]. FOXO family members represent one of several downstream direct substrates of PI3K/AKT signaling [85]. FOXO proteins phosphorylated by AKT are translocated from the nucleus to the cytoplasm and degraded via the ubiquitin-proteasome pathway [66]. FOXO family members function as tumor suppressors through upregulating cell cycle or apoptosis-related genes [86, 87]. For example, upregulation of miR-421 inhibits FOXO signaling pathway by directly targeting 3’-UTR of FOXO4 [88]. miR-96 confers the pathologically altered idiopathic pulmonary fibrosis (IPF) phenotype in response to collagen matrix via reducing FOXO3A and its targets p27, p21, and Bim [89]. The new relationship of FOXO1 with miRNA in prostate cancer was firstly reported by Wu et al [90]. In their study, overexpression of miR-370 increased cell proliferation by decreasing FOXO1 protein expression, accompanied with downregulation of the FOXO1 target genes (p21Cip1 and p27Kip1) and upregulation of cyclin D1. Followingly, Zhang and his colleagues showed that miR-370 represses FOXO1 expression via “seedless” 3’-UTR miRNA recognition elements in replantation tissues [91]. Interestingly, these factors also serve as effectors of the PI3K-AKT signaling pathway [92]. There is also interesting evidence suggesting that FOXO can regulate miRNA expression. For example, Wu et al showed that FOXO3A, as an important substrate of AKT, up-regulates the expression of pro-apoptotic inducer miR-30d [93]. A mechanism of cross-talk between Wnt/β-catenin and PI3K/AKT/FOXO signaling has been disclosed [94]. Liu et al revealed that FOXO3a activates the promoter that controls expression of the precursor RNA of miR-34b/34c, which in turn suppresses β-catenin expression and inhibits the expression of Wnt/β-catenin-target genes in prostate cancer [95].

Phosphatase and tensin homolog (PTEN) is a tumor suppressor which induces apoptosis and control cell growth, invasion, and angiogenesis through regulating several signaling pathways, including PI3K/AKT [96]. PTEN functions to convert phosphatidylinositol-3,4,5-triphosphate (PIP3) to a diphosphate product (PIP2) and consequently reverses the reactions catalyzed by PI3K, downregulating the kinase AKT [97]. Several mechanisms such as genetic mutation, promoter methylation and phosphorylation by the upstream kinase and targeted by miRNAs have been proposed for PTEN downregulation in various human cancers [98]. Moreover, many conserved miRNA target sites are found within the PTEN 3’-UTR, such as miR-21, miR-22, miR-214 and miR-126 [99–102]. Recently, miR-22 was reported to regulate the PTEN/AKT/FOXO1 pathway and together generate a feed-forward regulatory loop: miR-22 suppresses PTEN expression, leading to the activation of AKT activity, which in turn upregulates miR-22 transcription [103]. miR-486 was also reported to modulate PI3K/AKT signaling by targeting PTEN and blocking FOXO1 to benefit muscle metabolism (Figure 2E) [104].

The level of miR-145 level is observed to be significantly decreased in HCC, and its low expression is inversely associated with the abundance of insulin receptor substrate 1 (IRS1), a key mediator in oncogenic insulin-like growth factor signaling [105]. Recent evidence has shown that IRS1 is a strong inhibitor of FOXO1 via its AKT-mediated phosphorylation [106]. As a result, miR-145-induced IRS1 reduction decreases phosphorylation of AKT and sustains FOXO1 activity to suppress cancer cell growth. Similarly, ectopic miR-126 has been proposed to downregulate IRS1, reduce AKT signaling and inhibit cytosolic sequestration of FOXO1 in response to mitochondria-destabilizing stimuli involved in cancer induction and progression (Figure 2E) [107]. TGF-β/AKT/Snail axis is one of the important non-Smad parallel downstream pathways in EMT, in which TGF-β induces AKT activation and NF-κB nuclear translocation and then leads to elevated expression of the Snail transcription factor and reduced E-cadherin expression [108]. Moreover, TGF-β-induced EMT is partly regulated by miR-21 via the AKT pathway, while reactive oxygen species production further stimulates miR-21 synthesis [109]. Kallistatin is an endogenous plasma protein, which consists of two structural elements: active site and heparin-binding domain. This protein plays an important role in EMT-associated pathological processes, such as fibrosis and cancer [110]. Guo et al demonstrated for the first time that Kallistatin blocks TGF-β-induced miR-21 synthesis and ROS formation via its heparin-binding site, and conversely through its active site stimulates the synthesis of endothelial nitric oxide synthase, sirtuin 1 and FOXO1 (Figure 2F) [111].

Interestingly, FOXO3a has been shown to be one of the transcription factors for PTEN and a repressor of miR-21 expression [112, 113]. FOXO3a represses miR-21 expression by inhibiting the activation and DNA-binding activity of AP-1 [114, 115]. Aldose reductase (AR) has been reported to modulate expression of PI3K, NF-κB and AP-1 [116, 117]. Herein, under oxidative stress, researchers demonstrated that AR inhibition suppresses oncogenic miR-21 expression and upregulates PTEN and FOXO3a levels in colon cancer cells through PI3K/AKT/AP-1 pathway (Figure 2G) [115].

SIRT1 is an nicotinamide adenine dinucleotide (NAD^+)^-dependent class III histone deacetylase, which contributes to tumor progression by mediating promoter CpG island methylation and deacetylation of tumor suppressor proteins including FOXO family [118, 119]. Cancer-related SIRT1 overexpression is owing to the evasion of related miRNAs including miR-34a, miR-146b, miR-132/212 and miR-217 (Figure 2H) [120]. It has been reported that miR-34a inhibits SIRT1 expression to regulate cell apoptosis through a SIRT1-p53 pathway [121]. Zhao et al demonstrated for the first time that miR-34a induces endothelial progenitor cells senescence and impedes its angiogenesis by increasing FOXO1 acetylation following SIRT1 reduction [122]. In another report, miR-146b was found to bind directly to the 3’-UTR of SIRT1 and inhibit adipogenesis through SIRT1 downregulation and acetylation of FOXO1 [123]. Similar with miR-34a and miR-146b, miR-132 and miR-212, two tandemly expressed miRNAs, play vital roles in SIRT1-FOXO1 pathway. GnRH-induced expression of miR-132/212 was observed to regulate the decrease of SIRT1 deacetylase content, thus lead to an enhanced acetylation of FOXO1 [124]. Similarly, miR-217 inhibits SIRT1 expression by binding to the 3’-UTR of SIRT1, leading to loss of SIRT1 function on its major endothelial targets including FOXO1 and eNOS, finally promoting endothelial senescence [125]. SIRT2 is the most predominantly expressed in the brain via regulation of a variety of biological process, like aging, stress resistance and metabolism [126]. Date from Wang et al showed that decreased Sirt2 expression by miR-339 activates its targets such as NF-κB and FOXO1 through increasing their acetylation [127].

### FOXP subfamily and miRNAs

FOXP1, a tumor suppressor protein, is an essential participant in transcriptional regulatory network of B cell development [128]. B cell precursors contain pro-B cells, during which B cells rearrange IgH; pre-B cells, during which IgL is rearranged, and finally immature and mature B cells, expressing surface immunoglobulin IgM [129]. Constitutive expression of miR-34a makes a block in B cell development at the pro-B cell to pre-B cell transition, leading to a reduction in mature B cells. This block appeared to be mediated primarily by inhibited expression of the FOXP1, a direct target of miR-34a [130]. He et al showed that decreased miR-34a expression and increased FOXP1, p53, and BCL2 coexpression to predict a poor overall survival for gastric MALT lymphoma and DLBCL patients [131]. In chronic lymphocytic leukemia, high-level expression of miR-150 was observed to repress expression of FOXP1, which encodes proteins that enhance B-cell receptor signaling [132]. Another report showed that miR-206 promotes cardiomyocyte growth and survival in postnatal hearts through targeted degradation of the tumor suppressor FOXP1 [133]. Connective tissue growth factor (CTGF) is a multi-functional secreted protein which attenuates oral squamous cell carcinoma cellular invasion [134]. As a CTGF downstream, miR-504 is repressed by CTGF and carries out its ‘onco-miR’ function through targeting FOXP1 [135]. Cui and his colleagues identified FOXP1 as a direct target of miR-504 in glioma tumorigenesis and showed that miR-504/FOXP1 signaling significantly decreases cellular invasiveness and inhibits lymph node metastasis [136]. The biological mechanisms by which miR-504/FOXP1 signaling exerts opposing roles to tumorigenesis of different cancers need to be further elucidated.

Recently, FOXP2 is a widely concerned gene relevant to the human ability to develop language and speech [137]. The FOXP2 mRNA has an approximately 4-kb-long 3’-UTR, twice as long as its protein coding region, suggesting that FOXP2 can be regulated by miRNAs [138]. Recently, Clovis et al reported that miR-9 and miR-132 repress FOXP2 expression in mouse embryonic brain [139]. In a study, Shi and his colleagues provided evidence that miR-9 and miR-140-5p downregulate FOXP2 expression by targeting FOXP2 3’-UTR [140]. Similarly, other miRNAs (let-7a, miR-9, and miR-129-5p) are also found to inhibit FOXP2 expression in a dosage-dependent manner and target specific sequences in the 3’-UTR of FOXP2 during early cerebellum development [141]. Interestingly, substantial miRNAs (miR-9, miR-132, let-7a and miR-140-5p) are found to be lost in the striatum of mammals which is a region important for speech and language, where FOXP2 is expressed [142, 143]. In breast cancer, miR-199a was also reported to repress FOXP2. Also, elevated miR-199a and depressed FOXP2 expression levels are found to be prominent features of malignant clinical breast cancer and are associated with poor survival [144]. In another report, Jia et al firstly reported that an increased expression of miR-190 led to downregulation of FOXP2 in GC cells [145].

FOXP3 is a master regulator of CD4^+^CD25^+^ regulatory T (T_REG_) cell development and the forced expression of FOXP3 converts CD4^+^CD25^−^ T cells into T_REG_ cells [146]. FOXP3^+^ T_REG_ cells limit pathogenic immune responses to self-antigens and foreign antigens [147]. In this study, it was found that FOXP3 regulates miR-7 and miR-155 in breast cancer cell lines, suggesting that aberrant expression of miRNAs may be an important influence of FOXP3-deficency in primary tumor cells. There exists highly expressed miR-155 in regulating T_REG_ cell possibly for the reason that FOXP3 binds to the intron within the DNA sequence encoding the miR155 precursor mRNA, Bic [148]. Lu and his colleagues also found that FOXP3 facilitates miR-155 expression. Moreover, miR-155 deficiency in T_REG_ cells results in increased suppressor of cytokine signaling 1 expression in response to limiting amounts of interleukin-2 [149–151]. Huang et al showed that FOXP3 downregulates miR-142-3p to keep the adenylyl cyclase (AC) 9 / cAMP pathway active in T_REG_ cells [152]. In addition, miR-138 is an upstream regulator of FOXP4 and directly regulates FOXP4 expression in NSCLC [153]. Similarly, it was also found that miR-338-3p inhibits HCC cell growth by targeting FOXP4 [154].

### Other subfamilies and miRNAs

FOXF2 has been described to promote organ development, extracellular matrix (ECM) synthesis and epithelial-mesenchymal interaction [155]. This protein can be activated by hedgehog signaling and subsequently limit Bmp and Wnt signaling [156]. miR-182-induced downregulation of FOXF2 partially accounts for the increased activity of β-catenin signaling suggesting a potential mechanism underlying an miR-182/FoxF2 link contributing to CRC development [157]. Also, miR-519a promotes proliferation and inhibits apoptosis of HCC cells by targeting FOXF2 [158].

FOXG1 (formerly Brain Factor 1, BF-1) exerts important roles in the development of the telencephalon, the dorsal region of which develops into the neocortex in mammals [159]. The high conservation of the 3’-UTR across FOXG1 orthologues makes this region to maintain certain sequences including miRNA binding sites. miR-9 and miR-33 are the most possible candidate miRNAs due to their conservation and their location in flanking regions of low secondary structure stability [160]. The two miRNAs down-regulate FOXG1 expression, relieving the repression of neural genes by FOXG1 during forebrain development [160, 161].

FOXN1 is selectively expressed in skin epithelial cells and thymus, where it functions via molecular targets, including scf, ccl25, cxcl12 and dll4, to balance growth and differentiation. What's more, FOXN1 is essential for the transition from the initial epithelial thymic anlage to a functional cortical and medullary thymic epithelial cells (TECs) meshwork [162]. Researchers also showed that FOXN1 mutation generates thymic dysgenesis, causing primary T-cell immunodeficiency and leading to a hairless “nude” phenotype in both mice and humans for the reason of defective TECs [163]. A recent study has shown that FOXN1 is a target of miR-18b and miR-518b, and their silencing leads to FOXN1 up-regulation and epithelial lineage development in NT2/D1 and H9 human embryonic stem (hES) cells [164]. Yuan et al demonstrated that ectopic activation of miR-22 results in hair loss due to the repression of the hair keratinocyte differentiation program and pointed out that miR-22 directly represses myriad transcription factors upstream of phenotypic keratin genes, including FOXN1 [165]. miR-205 is necessary to maintain normal levels of FOXN1 in TECs, which in turn transcriptionally regulates ccl25 and scf [166].

FOXJ2 protein is able to bind to the E-cadherin promoter and induce its expression in human hepatoma, breast cancer and cervix carcinoma [167, 168]. miR-34a is implicated in cell differentiation pathways from stem cell and precursor populations [169]. Shear stress can induce the expression of miR-34a, which targets FOXJ2 to promote endothelial progenitor cells differentiation [170]. FOXJ3 (key transcription factors of mitochondrial biogenesis) is a newly identified transcription factor which upregulates Mef2c expression [171]. It was also observed that miR-494 regulates mitochondrial biogenesis by inhibiting FOXJ3 expression [172]. Meanwhile, overexpression of miR-27b provokes a decrease of mitochondria content and diminishes expression of FOXJ3 both at mRNA and protein levels by directly targets the 3’-UTR of FOXJ3 [173]. Furthermore, a recent study showed that miR-517a-3p accelerates lung cancer cell proliferation, migration and invasion through inhibition of FOXJ3 [174, 175].

FOXQ1 has been reported to function as an oncogene in various cancer types. Previous study has demonstrated that miR-506 inhibits proliferation and EMT of cervical cancer cells by targeting FOXQ1, suggesting that the miR-506/FOXQ1 axis plays an important role in the pathogenesis of cervical cancer [176]. Meanwhile, miR-124 and miR-506 function as a tumor-suppressive genes in nasopharyngeal carcinoma and their suppressive effects are mediated chiefly by repressing FOXQ1 expression [177, 178].

## The MICRORNA/FOX SIGNALINGS IN CANCER TREATMENT

The important roles of FOX transcription factors in tumor development ensure their usefulness in therapeutic interventions for human cancers. Data from recent studies suggest that FOX proteins are indirect targets of several widely used cancer drugs. For example, tyrosine kinase inhibitors (lapatinib and gefitinib) and monoclonal antibody (trastuzumab) have been shown to function by activating FOXO and by indirectly repressing FOXM1 via inhibiting PI3K/AKT signaling [179–181]. FOX proteins are regulated by miRNAs, and potentially their expression can therefore be targeted by RNA interference. After being demonstrated to be important regulators of FOX proteins in cancer cells, miRNAs are now being explored as potential targets for cancer molecular therapy. Correcting miRNA deregulation by either antagonizing or restoring miRNA function may provide a therapeutic insight [182]. Conceptually similar to other inhibitory therapies, miRNA antagonists are single-stranded RNA molecules which are 21-23 nucleotides in length and complementary to mature target miRNAs [182, 183]. On the other hand, tumor suppressor miRNA mimics are reintroduced into the cells to restore loss of function of the target genes [184]. The biggest obstacle when using miRNA mimetics is their delivery to the related tissues. Resolving the problem of miRNA delivery is essential for the use of miRNAs in cancer treatment. In 2007, Valadi et al found that exosomes contain miRNAs. They revealed that miRNAs derived from noncancerous cells can be transferred to cancerous cells, then inhibiting tumor cell proliferation [185]. Furthermore, researchers observed no overt side effects on exosome-mediated gene delivery [186, 187]. These studies indicate that miRNAs which specifically target kinds of FOX proteins can be loaded into exosomes and then be transported to cancer cells and therefore may be developed as possible anticancer drugs.

## CONCLUSIONS AND PERSPECTIVES

Findings from recent studies point out that FOX transcription factors might hold the key to significant progress in cancer treatment. For example, FOM1 expression is upregulated in malignancies, but it is barely expressed in non-diving normal cells, as a result indicating specificity for cancer cells in a targeted therapeutic approach [49] [11].Besides, the blooming research on FOX regulation, notably by posttranslational modification proves the importance and complexity of this family in diverse physiological and pathological conditions, including cancer. As demonstrated by numerous publications, a wide range of miRNAs can directly participate in the FOX signaling pathway forming positive or negative sophisticated feedback loops [74]. Mostly, malignant tumors have unique miRNA expression patterns that differentiate normal tissue from tumors. The unique miRNA may serve as a molecular biomarker for cancer diagnosis, tumor stage, prognosis, and prediction of cancer therapeutic responses [12].This new class of biomarkers can complement existing conventional markers, including antigens, metabolites and mRNA transcripts in some ways. Besides, miRNA inhibitors and miRNA mimics have become powerful standard tools. This is reflected in recent attempts to introduce safe and efficient miRNA-based and miRNA-targeted therapeutic approach. However, it still requires a much more in-depth investigation of the role of miRNA in modulating cancers before their true potential can be realized.

The most noticeable field in cancer research is the delineation of implementation of cancer therapeutics and tumorigenic pathways. The fact that many deregulated miRNAs potentially intermediate in the PI3K/AKT/FOXO signal pathways emphasizes the importance of miRNA and FOX coordinated pathways in ensuring tissue homeostasis during biological process[23].The miRNA-FOX signaling pathway may have a promising role in inhibition of cancer initiation and progression, and miRNAs that specifically target unique FOX can be used as anticancer drugs. However, firstly we must successfully identify the miRNAs that can effectively regulate FOX expression and develop simple, efficient methods of delivering miRNAs to cancer cells before using FOX- targeting miRNAs as anti-cancer drugs.

In this review, we have provided an overview on the current state of the literature describing the interactions between miRNAs and FOX transcription factors. Abnormal posttranscriptional regulation of FOX is often linked to various disease states including cancers. Future research into the importance and complexities of posttranscriptional regulatory interactions between microRNAs and FOX transcription factors will bring new knowledge about the mechanisms of disease processes and open novel ways towards therapeutic intervention.
